# Risk of Death from Prostate Cancer with and without Definitive Local Therapy when Gleason Pattern 5 is Present: A Surveillance, Epidemiology, and End Results Analysis

**DOI:** 10.7759/cureus.1453

**Published:** 2017-07-10

**Authors:** Jonathan Frandsen, Andrew Orton, Dennis Shrieve, Jonathan Tward

**Affiliations:** 1 Radiation Oncology, University of Utah Huntsman Cancer Hospital

**Keywords:** gleason score, prostate neoplasm, radiation therapy, combined modality treatment

## Abstract

Purpose

The purpose is to evaluate the patterns of care and comparative effectiveness for cause-specific and overall survival of definitive local treatments versus conservatively managed men with a primary or secondary Gleason pattern of 5.

Methods and materials

Patients diagnosed from 2004 to 2012 with a primary or secondary Gleason pattern of 5 N0M0 prostate cancer were extracted from the Surveillance, Epidemiology, and End Results (SEER) database. Kaplan-Meier and Cox regression analyses were used to estimate the survival.

Results

We identified 20,560 men. Median age and follow-up were 68 years and 4.33 years, respectively. At eight years, cause-specific survival (CSS) was 86.6% and 57.4% of those receiving and not receiving definitive local treatments, respectively. For CSS multivariate analysis, the following were significant: age, race, insurance status, total Gleason Score, T-stage, and type or omission of definitive local treatments. Compared to prostatectomy alone, men not undergoing definitive local treatments had the highest risk of death (HR: 6.07; 95% CI: 5.19-7.10). Those undergoing external beam radiotherapy alone (HR: 2.11; 95% CI: 1.80-2.48) were also at elevated risk of death. The number needed to treat (NNT) to prevent a prostate cancer death at eight years was three persons.

Conclusions

Death from prostate cancer with a primary or secondary Gleason pattern of 5 histology without definitive local treatment is high. In this hypothesis-generating study, we found that men with a limited life expectancy (less than eight years) and non-metastatic Gleason pattern of 5 disease may benefit from definitive local treatments. Given the high mortality in men with a Gleason pattern of 5, combined modality local therapies and consideration of chemotherapies may be warranted.

## Introduction

Men diagnosed with locally advanced prostate cancer have improved survival for definitive local therapy (DLT) with radiation treatment over androgen deprivation therapy (ADT) alone. In both the Canadian and Scandinavian randomized trials, the addition of radiation therapy to androgen deprivation was associated with an 8-10% overall survival (OS) benefit at 7-10 years [1-2]. With surgery, the Prostate Cancer Intervention Versus Observation Trial (PIVOT) (randomizing men to surgery versus conservative management) did not show a survival benefit in its initial report. However, the subset of men with high-risk cancers and those with Gleason scores (GS) >7 showed improved OS and cause-specific survival (CSS) over those in the conservative management group [3]. These randomized studies, however, included prostate cancers with a wide spectrum of T stages, GS, and prostate-specific antigen (PSA) values.

GS is an internationally recognized method for assigning a histological grade to prostate cancer. It is scored on a scale of 1-5, with scores closer to 5 representing higher grade disease. When GS is reported, two scores are assigned to cancer, the primary histological pattern identified and the secondary pattern. These two scores are commonly summed together to give a total GS [4-10]. A primary or secondary Gleason pattern of 5 (GP5) has been associated with an especially poor prognosis. Several studies have quantified the risk of biochemical failure or CSS and OS in this population [4-10]. However, most of these studies have limited their analyses to the outcomes of one type of therapy (or combination therapy). The purpose of this study is to evaluate the patterns of care and comparative effectiveness for CSS and OS of various routinely used definitive treatments over those of conservatively managed subjects in the especially high-risk group of men with a primary or secondary GP5.

## Materials and methods

The Surveillance, Epidemiology, and End Results (SEER) Program is an authoritative source of information on cancer incidence and survival in the United States. The collected data from 18 cancer registries represent approximately 28% of the US population diagnosed with any cancer. With each case submitted to the registry, important data are recorded including demographics, primary tumor site, tumor morphology, stage at diagnosis, the first course of treatment, and follow-up for vital status. For this project, data from the November 2014 SEER submission were utilized, which includes treated patients from 1973 to 2012. Approval by an internal review board for our study was not required as all SEER database information is de-identified.

Case selection

Our study population included any patient diagnosed with prostate cancer in the database from 2004 to 2012. The year 2004 was chosen as this was the first year GS was reported in the database. We used SEER*Stat software for data extraction. Using a ‘case listing’ session, we identified our patient population by querying for men with prostate cancer with either a primary or secondary GP5. Men with positive lymph nodes or distant metastases were excluded. For each case listing, we requested all variables included in the SEER registry. Six cohorts were then created, defined by the type of DLT: (1) no DLT, (2) prostatectomy alone, (3) prostatectomy with external beam radiation therapy (EBRT), (4) EBRT with brachytherapy (BT), (5) EBRT alone, and (6) BT alone.

Data analysis

Pearson chi-square analyses were used to compare patient and tumor characteristics for categorical variables. For continuous variables, one-way analysis of variance (ANOVA) was employed. Kaplan-Meier methods were then employed to analyze CSS and OS. Univariate and multivariate survival analyses were performed using Cox proportional-hazards regression methods, stratified by age. Only variables that were significant in univariate analysis were included in the multivariate analysis (MVA). Significance was defined as a p-value of <0.05. These analyses were performed using the STATA 14.0 statistical package (College station, TX).

## Results

In total, 20,560 men were identified that met our selection criteria. See Table 1 for patient and tumor characteristics by treatment group. The median age for men not receiving DLT was 78 years. The median age for men receiving DLT was 66 years. The treatment group with the lowest median age was surgery with adjuvant EBRT, with a median age of 62 years. The median follow-up time for men not receiving DLT was 3.75 years. The median follow-up time for men receiving DLT was 4.58 years.

Men not receiving DLT were more likely to be Black or other races, less likely to be insured by non-Medicaid insurance, and more likely to have a higher GS. Men undergoing prostatectomy alone or prostatectomy with EBRT were more likely to be White, and more likely to have a known Medicare or private insurance. Men undergoing prostatectomy alone were more likely to have the least aggressive histology (GS 3+5) by a wide margin compared to other treatment groups (Table 1).

**Table 1:** Patient and tumor characteristics. BT: Brachytherapy; EBRT: External beam radiation therapy.

	No Therapy		Surgery Alone		EBRT + BT		BT Alone		EBRT		Surgery + EBRT		
	n = 5269		n = 7272		n = 1027		n = 370		n = 4949		n = 1681		p-value
	No.	%	No.	%	No.	%	No.	%	No.	%	No.	%	
Age													<0.0001
Median years	78		64		69		71		72		62		
Follow-up													
Median years	3.75		4.08		5.17		5.33		5.00		3.92		
Race													<0.0001
White	3958	77	5908	82	803	79	292	79	3823	78	1387	83	
Black	847	17	828	11	129	13	63	17	703	14	166	10	
Other	304	6	483	7	89	9	13	4	381	8	125	7	
Gleason Score													<0.0001
3 + 5	531	10	1762	24	116	11	65	18	488	10	166	10	
4 + 5	2747	52	3824	53	587	57	192	52	2753	56	1002	60	
5 + 3	228	4	405	6	44	4	27	7	217	4	70	4	
5 + 4	1054	20	1014	14	154	15	42	11	863	17	338	20	
5 + 5	699	13	261	4	126	12	43	12	624	13	105	6	
T Stage													<0.0001
T1a-c	2204	42	3662	50	417	41	177	48	1946	39	726	43	
T2a (NOS)	1996	38	2027	28	265	26	113	31	1565	32	427	25	
T2b	77	1	274	4	69	7	22	6	164	3	72	4	
T2c	468	9	748	10	129	13	33	9	595	12	225	13	
T3a (NOS)	195	4	272	4	99	10	15	4	382	8	74	4	
T3b	80	2	182	3	43	4	9	2	190	4	131	8	
T4	154	3	30	<1	4	<1	0	0	92	2	13	1	
Tx	95	2	77	1	1	<1	1	<1	15	<1	13	1	
Insurance													<0.0001
Insured	1835	35	4656	64	481	47	156	42	2234	45	1116	66	
Medicaid	178	3	190	3	28	3	7	2	176	4	48	3	
Uninsured	49	1	71	1	4	0	0	0	50	1	26	2	
Unknown	3207	61	2355	32	514	50	207	56	2489	50	491	29	

**Figure 1:**
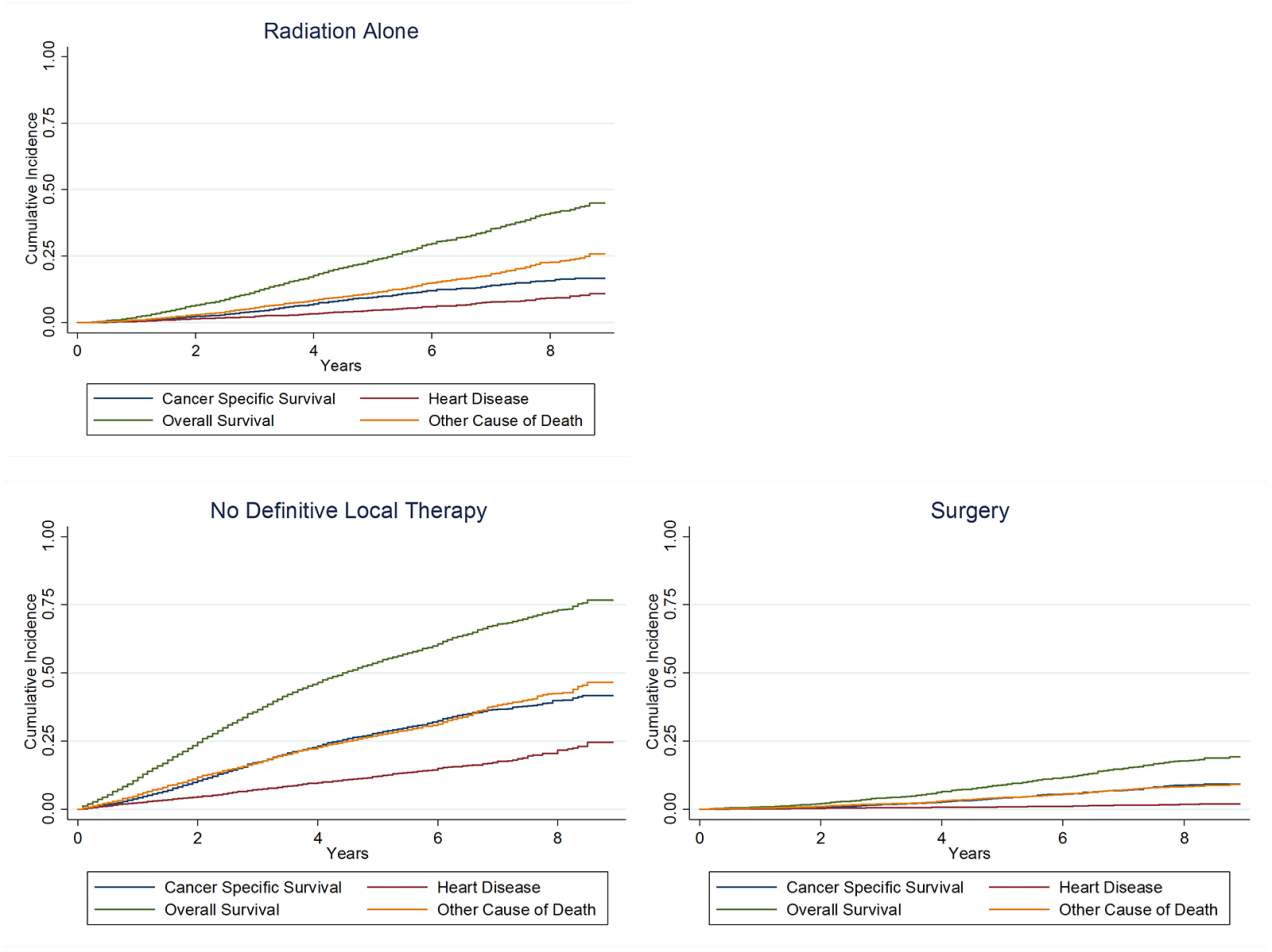
Cumulative incidence of the cause of death.

Men undergoing prostatectomy (with or without EBRT) were healthier than men receiving radiation treatments alone or men not receiving DLT as evidenced by rates of death from heart disease and other co-morbid conditions (Figure 1). At eight years cumulative incidence of death from heart disease was 1.3%, 7.9%, and 18.9% for men receiving prostatectomy, radiation alone, and no DLT, respectively (Figure 1).

**Figure 2:**
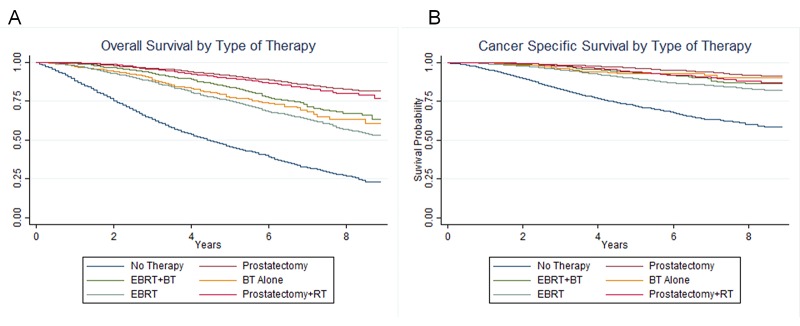
(A) Overall survival and (B) cancer-specific survival in men with prostate cancer with a primary or secondary Gleason pattern of 5. BT: Brachytherapy; EBRT: External beam radiation therapy.

For men not receiving DLT, OS at eight years was 66.8% versus 22.0% for those receiving and not receiving DLT, respectively (Figure 2). Cancer-specific survival at eight years was 86.6% for those receiving DLT and 57.4% for those not receiving DLT (Figure 2). Cancer-specific survival at three years was 97.2% for those receiving DLT and 82.9% for those not receiving DLT. Using the absolute difference in the cancer-specific survival, we calculated the number needed to treat (NNT) to save one life from prostate cancer death. The NNT to save a life from prostate cancer death was 3.4 at eight years and 7.0 at three years.

On univariate analysis for CSS, all of the following variables were found to be significant: age, race, insurance status, total GS, T-stage, and type or omission of DLT. All of these variables remained significant in the MVA (Table 2). Of note on the MVA, men not undergoing DLT performed worse than all types of DLT (HR: 6.07; 95% CI: 5.19-7.10). Men undergoing EBRT alone (HR: 2.11; 95% CI: 1.80-2.48) did worse than men undergoing prostatectomy alone (HR 1) or prostatectomy with EBRT (HR: 1.17; 95% CI: 0.91-1.50) (Table 2).

**Table 2:** Cox regression univariate and multivariate analysis of cancer-specific survival, stratified by age. BT: Brachytherapy; EBRT: External beam radiation therapy.

	Univariate		Multivariate	
Factor	aOR (95% CI)	p-value	aOR (95% CI)	p-value
Age	1.05 (1.04-1.05)	<0.001	1.01	
Race				
White	1		1	
Black	1.17 (1.05-1.31)	0.006	1.10 (0.97-1.23)	0.13
Other	0.61 (0.50-0.74)	<0.001	0.55 (0.45-0.68)	<0.001
Insurance				
Insured	1		1	
Medicaid	1.37 (1.04-1.80)	0.02	1.09 (0.83-1.44)	0.50
Uninsured	1.62 (1.05-2.50)	0.03	1.50 (0.90-2.33)	0.07
Gleason Score				
3 + 5	1		1	
4 + 5	2.60 (2.12-3.10)	<0.001	2.05 (1.71-2.47)	<0.001
5 + 3	1.99 (1.51-2.60)	<0.001	1.71 (1.30-2.25)	<0.001
5 + 4	4.15 (3.43-5.01)	<0.001	3.01 (2.48-3.65)	<0.001
5 + 5	5.58 (4.60-6.78)	<0.001	3.67 (3.00-4.49)	<0.001
Clinical T Stage				
T1a-c	1		1	
T2a and T2 NOS	1.20 (1.09-1.32)	<0.001	1.03 (094-1.14)	0.50
T2b	0.71 (0.53-0.96)	0.03	0.89 (0.66-1.20)	0.45
T2c	1.06 (0.92-1.22)	0.39	1.09 (0.95-1.26)	0.22
T3a and T3 NOS	1.28 (1.06-1.54)	0.009	1.25 (1.04-1.52)	0.02
T3b	1.68 (1.37-2.07)	<0.001	1.92 (1.56-2.37)	<0.001
T4	5.30 (4.33-6.47)	<0.001	3.02 (2.46-3.72)	<0.001
Type of Therapy				
No Therapy	8.11 (7.07-9.32)	<0.001	6.07 (5.19-7.10)	<0.001
Prostatectomy	1		1	
EBRT + BT	1.77 (1.38-2.29)	<0.001	1.47 ( 1.13-1.90)	0.004
BT Alone	1.62 (1.08-2.24)	0.02	1.41 (0.94-2.12)	0.10
EBRT	2.66 (2.28-3.10)	<0.001	2.11 (1.80-2.48)	<0.001
Prostatectomy + RT	1.53 (1.89-1.96)	0.001	1.26 (0.97-1.62)	0.08

**Figure 3:**
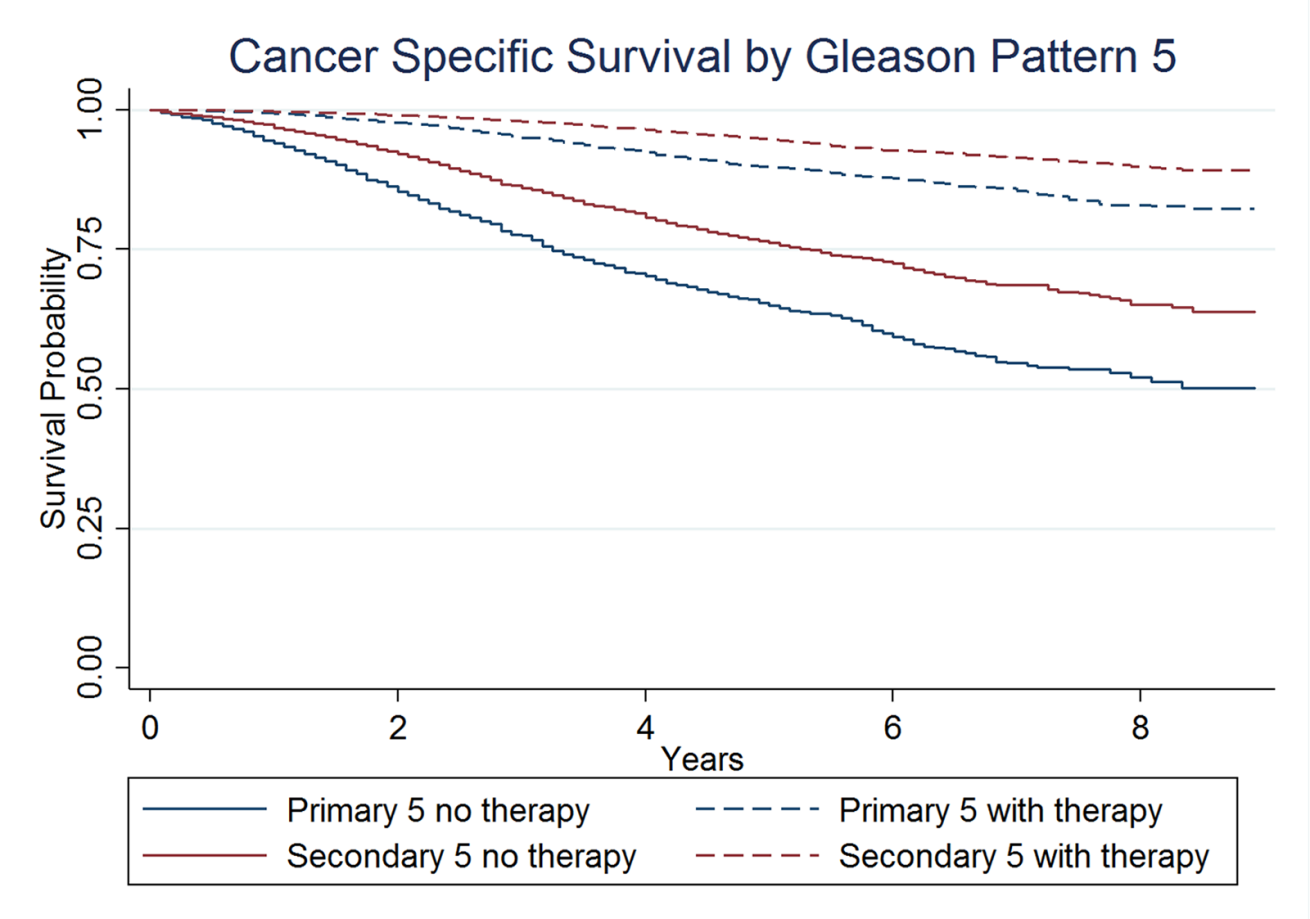
Cancer-specific survival by primary versus secondary Gleason pattern of 5, stratified by receipt of definitive local therapy.

For men with a primary GP5, CSS at eight years was 82.4% and 52.1% with and without DLT, respectively. For men with a secondary GP5, CSS at eight years was 89.9% and 64.8% with and without DLT, respectively (Figure 3). In men with a primary GP5, the NNT with DLT to prevent one prostate cancer death at eight years is 3.3 persons. In men with a secondary GP5, the NNT at eight years is 4.0 persons.

## Discussion

Men with a life expectancy of less than 10 years are often counseled to not undergo prostate cancer screening or definitive therapy [3]. We found that DLT was associated with a significant improvement in both OS and CSS within only a few years. In fact, only 7.0 and 3.4 persons need treatment with DLT to prevent a death from prostate cancer at three and eight years, respectively.

The observed benefit of DLT in our study was larger than noted in previous studies analyzing men in the more heterogeneous group with National Comprehensive Cancer Network (NCCN) or D’Amico high-risk, or locally advanced, prostate cancers [1-2, 11-12]. This is likely due to the fact that these men have a wider range of risk than those in our study due to the inclusion of men who meet high-risk definitions by PSA criteria and/or clinical T-stage criteria alone. A primary or secondary GP5 has been demonstrated to be one of the most prognostic risk factors for death from localized prostate cancer [7]. An alternative explanation for the difference between our study and those looking exclusively at high-risk cancers is that all men included in these previously completed studies were known to have received androgen deprivation therapy (ADT). Although it seems likely that most men in our study would have received ADT, the SEER database does not track the use of systemic therapies. If a substantial number of men in our study did not receive ADT in the “no DLT” cohort, one could hypothesize that it might exaggerate the benefit we observed for DLT versus no DLT. Nevertheless, there is evidence that primary ADT over delayed therapy does not improve OS or CSS, so this argument may be moot [13]. Conversely, it is possible that some of the men receiving radiation therapies in our study did not receive neoadjuvant, concurrent, and/or adjuvant ADT. If this were true, it would underestimate the benefit of radiation therapy, as numerous randomized trials have demonstrated an OS benefit to ADT with EBRT in the high-risk populations [14-16].

The average age of men not receiving DLT in our study was 78 years. According to the Social Security Life Expectancy Calculator, the average life expectancy of a 78-year-old male in the United States is 10.0 years [17]. Given the early survival benefit observed for DLT, elderly men in reasonably good health should at least be offered DLT. This recommendation is consistent with the NCCN prostate cancer treatment guidelines which recommend consideration of treatment in men with high-risk cancers even if they are asymptomatic and have a less than 6-year life expectancy [18]. The benefit of DLT was seen for men with both primary and secondary GP5 disease. While the benefit of DLT for men with a primary GP5 was larger than for men with secondary GP5, the benefit for a secondary GP5 was also large enough to justify the treatment of elderly men in good health.

Men undergoing prostatectomy alone or with EBRT correlated with better CSS on both univariate and multivariate analyses compared to all other modalities aside from BT alone. The comparison of various local therapies for prostate cancer using the SEER database is confounded by the omission of important prognostic factors, such as pretreatment PSA, receipt of ADT, and information about radiotherapy dose and field design. In addition, men undergoing prostatectomy (with or without EBRT) clearly had more favorable factors including younger age, a healthier population, better GS, race, and were more often privately or Medicare-insured. These factors are all associated with improved survival in prostate cancer [19-23]. Although we attempted to control these factors in our MVA, we could not control the important prognostic factors of ADT use and PSA, nor medical comorbidities of the underlying populations. Men in the prostatectomy cohorts were far less likely to die of non-prostate cancer deaths than those in the radiation groups (especially heart disease). This strongly implies that men in the non-prostatectomy cohorts were less healthy than those undergoing surgery, which biases the OS differences into favoring surgery. The cause of death data contained within the SEER registry is gathered from death certificates, which relies on providers accurately assessing the cause of death. As an example, if an elderly man were to die from a non-oncologic cause and happened to have metastatic prostate cancer, the practitioner completing the death certificate may interpret this as related to their oncologic diagnosis, although cancer may not have contributed. In this case, the DSS outcomes could also inappropriately favor surgery over radiations. Although we can argue why surgery may or may not be superior to radiations in this very high-risk cohort, in the absence of a contemporary randomized prospective trial comparing these modalities with modern dose-escalated radiotherapy and surgical techniques, we cannot know if the differences are real. What can be stated unequivocally, based on these data, is that DLT of any kind is superior to no DLT. Despite the omission of important clinical information in the SEER registry, it should be noted that EBRT alone performed worse than all the other local therapies analyzed. This is consistent with meta-analysis data and prospective randomized trial data which show EBRT to be inferior to combined modality treatments [24-25].

Given the highly aggressive nature of GP5 disease noted in our study, the addition of chemotherapy to this high-risk subset of patients might be indicated. Docetaxel has long been used in the metastatic, castrate-resistant setting, resulting in improved survival [26]. More recently, the Systemic Therapy in Advancing or Metastatic Prostate Cancer: Evaluation of Drug Efficacy (STAMPEDE) trial and the Chemohormonal Therapy versus Androgen Ablation Randomized Trial for Extensive Disease in Prostate Cancer (CHAARTED) demonstrated improved survival with the addition of Docetaxel to ADT in the castrate-sensitive phase of disease, chiefly in men with metastatic disease [27-28]. In men with non-metastatic, high-risk disease, RTOG 0521 demonstrated improved survival with the addition of Docetaxel [29]. The absolute difference in survival at four years in this study was 4% and was only significant by a 1-sided t-test (as opposed to the traditional 2-sided test), suggesting a marginal benefit. The study included men with a wide range of risk (GS of 7-10). One could hypothesize that men with the highest risk histology (primary or secondary GP5) may have the greatest improvement in survival. Since RTOG 0521 has been presented in abstract form but not yet published, we do not know if the GS 9 and 10 subsets benefitted more so than other cohorts.

## Conclusions

Despite the biases inherent in retrospective population-based studies, they provide a good understanding of what is happening in the real-world conditions of multiple providers with various differences in practice patterns, medical accessibility, and skills. In this hypothesis-generating analysis, DLT was associated with superior survival and the gains were realized within just a few years. This suggests that all men with a GP5 might benefit from DLT unless there are significant medical comorbidities that would prevent DLT from being delivered. Given the high mortality in this subset, combined modality therapies and consideration of chemotherapies may be warranted.

## References

[1] Warde P, Mason M, Ding K (2011). Combined androgen deprivation therapy and radiation therapy for locally advanced prostate cancer: a randomised, phase 3 trial. Lancet.

[2] Widmark A, Klepp O, Solberg A (2009). Endocrine treatment, with or without radiotherapy, in locally advanced prostate cancer (SPCG- 7/SFUO-3): an open randomised phase III trial. Lancet.

[3] Wilt TJ, Brawer MK, Jones KM (2012). Radical prostatectomy versus observation for localized prostate cancer. N Engl J Med.

[4] Hashine K, Yuasa A, Shinomori K (2011). Tertiary Gleason pattern 5 and oncological outcomes after radical prostatectomy. Jpn J Clin Oncol.

[5] Trock BJ, Guo CC, Gonzalgo ML (2009). Tertiary Gleason patterns and biochemical recurrence after prostatectomy: proposal for a modified Gleason scoring system. J Urol.

[6] Stock RG, Cesaretti JA, Hall SJ (2009). Outcomes for patients with high-grade prostate cancer treated with a combination of brachytherapy, external beam radiotherapy and hormonal therapy. BJU Int.

[7] Sabolch A, Feng FY, Daignault-Newton S (2011). Gleason pattern 5 is the greatest risk factor for clinical failure and death from prostate cancer after dose-escalated radiation therapy and hormonal ablation. Int J Radiat Oncol Biol Phys.

[8] Vis AN, Roemeling S, Kranse R (2007). Should we replace the Gleason score with the amount of high-grade prostate cancer?. Eur Urol.

[9] Nanda A, Chen MH, Renshaw AA (2009). Gleason Pattern 5 prostate cancer: further stratification of patients with high-risk disease and implications for future randomized trials. Int J Radiat Oncol Biol Phys.

[10] Cheng L, Koch MO, Juliar BE (2005). The combined percentage of Gleason patterns 4 and 5 is the best predictor of cancer progression after radical prostatectomy. J Clin Oncol.

[11] Bekelman JE, Mitra N, Handorf EA (2015). Effectiveness of androgen-deprivation therapy and radiotherapy for older men with locally advanced prostate cancer. J Clin Oncol.

[12] Lu-Yao GL, Albertsen PC, Moore DF (2008). Survival following primary androgen deprivation therapy among men with localized prostate cancer. JAMA.

[13] Potosky AL, Haque R, Cassidy-Bushrow AE (2014). Effectiveness of primary androgen-deprivation therapy for clinically localized prostate cancer. J Clin Oncol.

[14] Pilepich MV, Caplan R, Byhardt RW (1997). Phase III trial of androgen suppression using goserelin in unfavorable-prognosis carcinoma of the prostate treated with definitive radiotherapy: report of Radiation Therapy Oncology Group Protocol 85-31. J Clin Oncol.

[15] Bolla M, Collette L, Blank L (2002). Long-term results with immediate androgen suppression and external irradiation in patients with locally advanced prostate cancer (an EORTC study): a phase III randomised trial. Lancet.

[16] D’Amico AV, Chen MH, Renshaw A (2008). Risk of prostate cancer recurrence in men treated with radiation alone or in conjunction with combined or less than combined androgen suppression therapy. J Clin Oncol.

[17] (2015). Social security online calculator. https://www.socialsecurity.gov/cgi-bin/longevity.cgi. Accessed.

[18] Comprehensive Cancer (2015). Prostate cancer. Accessed.

[19] Marlow NM, Halpern MT, Pavluck AI (2010). Disparities associated with advanced prostate cancer stage at diagnosis. J Health Care Poor Underserved.

[20] Polednak AP (2003). Black-White differences in survival from late-stage prostate cancer. Ethn Dis.

[21] Gross CP, Smith BD, Wolf E (2008). Racial disparities in cancer therapy: did the gap narrow between 1992 and 2002?. Cancer.

[22] Polednak AP (1998). Prostate cancer treatment in black and white men: the need to consider both stage at diagnosis and socioeconomic status. J Natl Med Assoc.

[23] Walker GV, Grant SR, Guadagnolo BA (2014). Disparities in stage at diagnosis, treatment, and survival in nonelderly adult patients with cancer according to insurance status. J Clin Oncol.

[24] Grimm P, Billiet I, Bostwick D (2012). Comparative analysis of prostate-specific antigen free survival outcomes for patients with low, intermediate and high risk prostate cancer treatment by radical therapy. Results from the Prostate Cancer Results Study Group. BJU Int.

[25] Morris WJ, Tyldesley S, Pai HH (2015). ASCENDE-RT*: A multicenter, randomized trial of dose-escalated external beam radiation therapy (EBRT-B) versus low-dose-rate brachytherapy (LDR-B) for men with unfavorable-risk localized prostate cancer. J Clin Oncol.

[26] Tannock IF, de Wit R, Berry WR (2004). Docetaxel plus prednisone or mitoxantrone plus prednisone for advanced prostate cancer. N Engl J Med.

[27] James ND, Sydes MR, Mason MD (2015). Docetaxel and/or zoledronic acid for hormone-naïve prostate cancer: first overall survival results from STAMPEDE (NCT00268476). J Clin Oncol.

[28] Sweeney CJ, Chen YH, Carducci M (2015). Chemohormonal therapy in metastatic hormone-sensitive prostate cancer. N Engl J Med.

[29] Sandler HM, Hu C, Rosenthal SA (2015). A phase III protocol of androgen suppression and 3DCRT/IMRT versus AS and 3DCRT/IMRT followed by chemotherapy with docetaxel and prednisone for localized, high-risk prostate cancer (RTOG 0521). J Clin Oncol.

